# Offering extended use of the contraceptive implant via an implementation science framework: a qualitative study of clinicians’ perceived barriers and facilitators

**DOI:** 10.1186/s12913-024-10991-4

**Published:** 2024-06-03

**Authors:** Nicole Rigler, Gennifer Kully, Marisa C. Hildebrand, Sarah Averbach, Sheila K. Mody

**Affiliations:** 1 School of Medicine, University of California San Diego, San Diego, CA USA; 2 Division of Complex Family Planning, Department of Obstetrics, Gynecology and Reproductive Sciences, University of California San Diego, 9300 Campus Point Dr. MC 7433, La Jolla, San Diego, CA USA; 3 Center on Gender Equity and Health, University of California, San Diego, CA USA

**Keywords:** Contraceptive implant, Long-acting contraception, Long-acting reversible contraception, LARC, Extended use, Contraceptive access, Implementation science, Consolidated framework for implementation research, Off-label use

## Abstract

**Background:**

The etonogestrel contraceptive implant is currently approved by the United States Food and Drug Administration (FDA) for the prevention of pregnancy up to 3 years. However, studies that suggest efficacy up to 5 years. There is little information on the prevalence of extended use and the factors that influence clinicians in offering extended use. We investigated clinician perspectives on the barriers and facilitators to offering extended use of the contraceptive implant.

**Methods:**

Using the Consolidated Framework for Implementation Research (CFIR), we conducted semi-structured qualitative interviews. Participants were recruited from a nationwide survey study of reproductive health clinicians on their knowledge and perspective of extended use of the contraceptive implant. To optimize the diversity of perspectives, we purposefully sampled participants from this study. We used content analysis and consensual qualitative research methods to inform our coding and data analysis. Themes arose deductively and inductively.

**Results:**

We interviewed 20 clinicians including advance practice clinicians, family medicine physicians, obstetrician/gynecologist and complex family planning sub-specialists. Themes regarding barriers and facilitators to extended use of the contraceptive implant emerged. Barriers included the FDA approval for 3 years and clinician concern about liability in the context of off-label use of the contraceptive implant. Educational materials and a champion of extended use were facilitators.

**Conclusions:**

There is opportunity to expand access to extended use of the contraceptive implant by developing educational materials for clinicians and patients, identifying a champion of extended use, and providing information on extended use prior to replacement appointments at 3 years.

**Supplementary Information:**

The online version contains supplementary material available at 10.1186/s12913-024-10991-4.

## Background

The etonogestrel contraceptive implant is currently approved by the U.S. Food and Drug Administration (FDA) for 3 years of continuous use for the prevention of pregnancy [1]. However, there is evidence to support its use for up to 5 years while maintaining a low risk of pregnancy [2–4]. The off-label use of the contraceptive implant past its FDA-approved duration and up to 5 years is known as extended use. Importantly, the FDA supports off-label use of marketed drugs and medical devices so long as there is strong relevant published evidence [5]. Off-label use such as extended use of the contraceptive implant is common with many other reproductive devices and medications, including misoprostol for labor induction, the copper intrauterine device (IUD) for emergency contraception, and, prior to its recent FDA-approval for extended use, the 52 mg levonorgestrel (LNG) IUD for pregnancy prevention. The 52 mg LNG IUD was previously FDA-approved for 5 years, however strong published evidence demonstrated longer efficacy up to 8 years, leading clinicians to counsel on extended use and eventually contributing to updated federal guidelines [6, 7].

Though there are clinicians who counsel patients on extended use of the contraceptive implant, many patients still undergo implant replacement after only 3 years of use [8, 9]. Continuation rates of the contraceptive implant after 1 and 2 years of use is estimated to be at 81.7% and 68.7%, with the most common reason for early discontinuation prior to 3 years being changes to bleeding pattern [10–13]. Ali et al. report the most common reasons that patients decided to stop implant use in years 4 and 5: unspecified personal reasons, desired fertility, bleeding problems, and other medical reasons [4]. Additionally, a recent nationwide, web-based survey amongst a diverse group of reproductive health clinicians investigated the barriers and facilitators regarding extended use of the contraceptive implant up to 5 years [14]. The most common barriers found in the study were provider concerns about pregnancy risk and the current FDA approval for only 3 years of use. The key facilitators included strong published evidence supporting extended use and patient and clinician education on extended use. Other than these studies, the patient and clinician factors that facilitate and hinder widespread implementation of extended use of the contraceptive implant have not been explored.

Increasing implementation of extended use of the contraceptive implant across practice settings may decrease unnecessary procedures, devices, healthcare visits, and could improve access to, and satisfaction with, the contraceptive implant. Long-acting reversible contraceptive (LARC) methods such as the contraceptive implant and LNG IUD have significantly higher continuation and approval rates and are more efficacious at preventing pregnancy than non-LARC methods such as oral contraceptive pills and depot medroxyprogesterone acetate injection [12, 15, [16]. Given the continued high rates of unintended pregnancies in the United States and the consequential increase in healthcare costs and poor outcomes secondary to pregnancy complications, efficacious pregnancy prevention is an important public health objective and cost-saving measure [17].

Using a qualitative approach guided by an implementation science framework, the Consolidated Framework for Implementation Research (CFIR), [18] we sought to explore clinician perspectives on extended use of the contraceptive implant up to 5 years as well as the perceived barriers and facilitators for clinicians to offer extended use.

## Methods

We conducted semi-structured interviews with 20 clinicians including obstetrics and gynecology generalists, family medicine physicians, complex family planning sub-specialists, and advanced practice clinicians. We recruited interview participants from a nationwide, web-based survey that assessed the prevalence of extended use of the contraceptive implant [17]. This study recruited respondents through email listservs for the Fellowship in Complex Family Planning, the Ryan Residency Training in Family Planning Program, women’s health nurse practitioners, and family medicine physicians, as well as private social media groups for obstetrician-gynecologists. The total reach of the survey was unknown, however, the study had a survey completion rate of 66.6% (*n* = 300/450). Of the 300 completed surveys, 290 respondents indicated their interest in being interviewed (96.7%).

Among the survey respondents, we invited 24 clinicians to participate in interviews, yielding an 83.3% response rate. We selectively recruited interview participants to enrich our sample, specifically focusing on clinician type, practice setting, and region of practice within the United States (U.S.). We also selected interview participants based on whether they always, sometimes, or never counsel on extended use to investigate a broad range of perspectives. For this study, offering extended use is defined as counseling on use past the current FDA-approved duration of 3 years and up to 5 years of use. Offering extended use can occur at any clinical encounter, including insertion appointments, replacement and removal appointments at or before 3 years, and general reproductive health appointments. Clinicians who always offer extended use were defined as those who counsel on extended use to patients who are considering or currently have the contraceptive implant. Clinicians who sometimes offer extended use were defined as those who counsel on extended use, but only to particular patients based on patient-specific factors such as body mass index or insurance coverage. Clinicians who never offer extended use were defined as those who never counsel on use of the contraceptive implant past 3 years of use.

The interview guide was created utilizing an implementation science framework that identifies factors for effectively enacting interventions [18]. The Consolidated Framework for Implementation Research (CFIR) is organized into 5 major domains: characteristics of the intervention, individual characteristics, inner setting, outer setting, and the process of implementation. The first domain, intervention characteristics, relates to the inherent qualities of the intervention, such as pharmacologic properties and side effects of the contraceptive implant when used up to 5 years. Individual characteristics relates to the roles and characteristics of individual patients and clinicians interacting with the intervention, such as educational background and type of insurance coverage. The inner setting domain assesses the internal setting in which an intervention will be implemented (i.e., clinic type, culture, and policies). The broader context in which an intervention will be implemented, including national policies and social norms is evaluated within the outer setting domain. Finally, the process of implementation domain explores the activities and strategies used to implement the intervention, such as educational materials or clinician and staff trainings on extended use.

We designed the interview guide around these specific domains with questions that aimed to identify targeted strategies to support successful implementation. The complete interview guide is in Appendix A. The interview guide was designed with input from clinicians who regularly prescribe contraception, including extended use of the contraceptive implant, as well as CFIR and implementation science experts. The Human Research Protection Program at our institution approved the study.

A single research team member conducted semi-structured interviews via secure video conference between July and August 2021. Interview participants provided informed consent. All participants were asked a full set of open-ended questions based on the interview guide, with focused follow-up questions to further investigate potential themes or to clarify points. All interviews were audio recorded, then transcribed. For data analysis, we used a content analysis approach to identify concepts and patterns within the dataset [19]. Themes arose deductively and inductively, with deductive themes identified from the CFIR domains and inductive themes arising from interview insights. Consensual qualitative research methods informed both our data analysis and coding process [20]. Three authors were involved in the thematic coding of the transcripts. Initially, 5 transcripts were independently coded then checked for inter-coder reliability. Any disagreements were discussed, and a consensus was achieved. The remaining transcripts were then coded by one of the three authors. Once all interviews were coded, major themes and representative quotes were identified. The research team utilized ATLAS.ti for analysis [21].

## Results

Between July and August 2021, we interviewed 20 clinicians from a variety of clinical settings, regions, and women’s health professions, achieving the intended diversity of perspectives (Table 1). Among participants, 7 (35.0%) always, 8 (40.0%) sometimes, 5 (25.0%) never offer extended use of the contraceptive implant (Table 2).

**Table 1:** Demographics of reproductive health clinicians

	n (%) *N* = 20
Clinician Type	
Family Medicine Physician	5 (25)
General OB/GYN Physician	6 (30)
Advanced Practice Clinician	6 (30)
Complex Family Planning Specialist	3 (15)
Clinical Setting^a^	
Academic or University-Affiliated	9 (45)
Community	6 (30)
Private Practice	3 (15)
Other^b^	2 (10)
Region of Practice	
West Coast	6 (30)
East Coast	3 (15)
Southwest	3 (15)
Southeast	4 (20)
Midwest	4 (20)
Age	
27-29 years	1 (5)
30-39 years	10 (5)
40-49 years	8 (40)
50+ years	1 (5)
Gender	
Female	18 (90)
Male	2 (10)
Race	
White	14 (70)
Black or African American	1 (5)
Asian/Pacific Islander	3 (15)
Latinx/Mexican American	1 (5)
Other or multiracial	1 (5)

^a^Participants self-reported their clinical setting. In general, community clinics are those affiliated with non-academic hospital systems or non-profit organizations. Private practices are clinics that are owned and run by clinicians and are usually not affiliated with a hospital system

^b^Included a military hospital and a multispecialty, multidisciplinary group practice

**Table 2:** Barriers and facilitators to offering extended use of the contraceptive implant

**Patient Level**	
** CFIR Domain**	**Barrier to Implementation**
Outer Setting	Patient concern about pregnancy risk
	Patient concern about bleeding profile changes
	Patient preference for removal/ replacement because already in clinic for removal/replacement
	Patient concern about future insurance coverage
** CFIR Domain**	**Facilitator to Implementation**
Inner Setting	Educational materials for patients (particularly handouts)
**Clinician Level**	
** CFIR Domain**	**Barrier to Implementation**
Outer Setting	Current FDA approval for 3 years Provider concern about liability in the case of unintended pregnancy
Inner Setting	Policies of my healthcare system Decreased incentive to delay procedures due to decreased RVU generation
	Decreased cohesion amongst clinicians within the same practice
** CFIR Domain**	**Facilitator to Implementation**
Outer Setting	Public health benefit of decreased costs and healthcare appointments Strong evidence supporting duration of use beyond FDA approval
Inner Setting	Colleagues offering extended use
	Provider training
	Easy rescheduling of replacement/ removal
Process of Implementation	A champion of extended use at my institution who is available to provide training and answer questions

### Characteristics of the intervention

We found that changes to bleeding pattern in or after the third year of use was a barrier to clinicians offering extended use of the contraceptive implant. The participants in this study noted that perceived increases in the irregularity or frequency of a patient’s bleeding makes extended use of the implant difficult for patients to accept. One clinician noticed that some patients correlate changes in their bleeding pattern with a perceived decrease in the efficacy of their implant:

"People who do start noticing changes in bleeding pattern […] [and] associating that with, ‘Oh, my implant is wearing out or becoming expired. I need to get this changed out."

-Complex Family Planning Specialist, Southwest, Academic Setting, sometimes offers extended use

The same clinician discussed that more research on bleeding patterns in the extended use period and potential treatments for implant-associated irregularities could be a facilitator of extended use:

"For bleeding, I think it would be awesome if there is a research study, looking at use of OCPs [oral contraceptive pills] to manage bleeding near the end of the use of an implant or near that three-year mark,, […] So that we could give people… Honestly, either a natural history or a, ‘Here’s how you can manage that if you do want to keep using your implant longer.’"

- Complex Family Planning Specialist, Southwest, Academic Setting, sometimes offers extended use

Information on the bleeding pattern in years 4 and 5 of use and how clinicians can address irregular bleeding during implant use may increase acceptability of extended use.

### Individual characteristics

We found that insurance impacts whether a clinician offers extended use:

"I do sometimes have patients saying, ‘I might be changing jobs or I’m going to be turning 27 or whatever.’ And so insurance is a barrier and so they’re like, ‘I want the new one while I still have this insurance.’"

- Family Medicine Physician, Midwest, Community Setting, sometimes offers extended use

Many participants agreed with this concept and stated that acceptability of extended use depends on a patient’s perception of their future insurance status. Clinicians observed that if a patient believes they will have coverage for a replacement or removal in the future, they are more likely to pursue extended use of their implant. Conversely, one clinician discussed how lack of current insurance coverage could be a facilitator of extended use:

"So, I would generally offer extended use to people that didn’t have insurance and would have to self-pay. I would like go through the data with them so they wouldn’t have to pay like $1,000 to get a new implant because it could work another year, or people that were concerned about changing side effects at that time."

- Obstetrician-Gynecologist, Southwest, Academic Setting, sometimes offers extended use

Overall, clinicians perceived that patients’ concerns about current and future insurance coverage may affect acceptance of extended use.

### Inner setting

This study found that having a champion of extended use at a clinician’s home or affiliate institution was a facilitator of extended use. Most clinicians in the study stated that it is or would be helpful to have someone who worked with them clinically that was knowledgeable on the data about extended use. When asked which factor would promote extended use of the implant the most, this clinician stated:

"…having a champion who is really ready to present the evidence, because the evidence can be there, but people don’t have time to read it. If it’s not brought to them, they’re not really going to know about it."

- Obstetrician-Gynecologist, West Coast, Community Setting, does not offer extended use

Potential champions identified were physicians, nurses, medical directors, or other clinicians in leadership positions, but participants generally believed that the position should be held by someone who is passionate about contraception, highly familiar with the specific setting, and knowledgeable about the clinical studies on extended use.

A barrier noted by a few participants was the effect of discordant counseling by different clinicians, sometimes within the same clinic, on acceptability of extended use:

"I mean, I guess like getting everyone on the same page, like in your practice can be a barrier. Especially in the practice I’ve been at, which like I said was in a state that was very litigious, so people weren’t always willing to like go outside guidelines that were… So getting your whole group on the same page so patients get like a more consistent message."

- Obstetrician-Gynecologist, Southwest, Academic Setting, sometimes offers extended use.

Participants discussed that it is important for clinician teams to relay a cohesive message to patients, especially in settings where patients may see multiple clinicians for their contraceptive care.

### Outer setting

Lack of FDA approval for extended use was identified as barrier by many clinicians, and some clinicians counseled patients only on the FDA-approved duration of the contraceptive implant:

"So, generally in our practice we don’t really talk about extended use. We say this is FDA approved for three years."

- Advanced Practice Clinician, Southeast, Community Setting, sometimes offers extended use.

Even clinicians who do offer extended use of the implant noted that off-label use can be confusing to patients, making it difficult to counsel on extended use:

"So I have patients all the time, who’ll say, ‘Well, what do you mean I can keep X, Y or Z in for an extra year?’ And I’ll say, ‘We have big studies that tell us that this is an okay thing to do.’ But that just feels weird. People don’t necessarily understand the role of the FDA or sort of how it works. And so it’s something like extended use just might be a really such a foreign concept. Right? It’s so far outside. But I think that there are also, there are lay outlets that cover this stuff. So it’s not that it’s impossible to access. It’s just that the patient has to be interested just like the provider has to be interested."

- Complex Family Planning Specialist, East Coast, Academic Setting, sometimes offers extended use.

Clinicians also observed that certain clinics must follow official guidelines without the flexibility to offer extended use, regardless of a clinician’s perspective or willingness to counsel on extended use. Interestingly, patient confusion as well as mistrust of the healthcare system may impact patient acceptability of extended use in the context of a three-year FDA-approved duration:

"The other thing is the FDA approval because the box says three years, but then like I tell people, you can take it out in five years. And then they don’t believe… Like who is right. Is it my doctor who’s getting in front of me right or the box, right?"

- Family Medicine Physician, West Coast, Community Setting, always offers extended use.

This clinician noted that a disconnect between a clinician’s counseling and prescription information may lead patients to be confused about the recommendation for extended use.

Another barrier mentioned by a few participants was provider concern about liability in the event of an unintended pregnancy. Participants discussed fear of both legal and interpersonal repercussions of unintended pregnancy after counseling on off-label use of a contraceptive device:

"Even though there’s a slim chance that a patient would get pregnant on Nexplanon [the contraceptive implant], I feel like if we were to say, ‘Yeah, you can use it beyond the four years,’ and they come up and they get pregnant, they’re that 1% chance that gets pregnant, I feel like there could be a little bit of blame laid on us if we were to tell them that they’re able to it beyond the three years when the label doesn’t say that yet."

- Advanced Practice Clinician, Southeast, Private Practice, does not offer extended use.

Some participants felt that they would “have no ground to stand on” in the event of a lawsuit (OBGYN Physician, Midwest, Private Practice), making them concerned about the possibility of increased liability in counseling on off-label use without FDA approval.

Interestingly, multiple clinicians also discussed abortion restrictions in the United States as influencing patients in their decision to pursue extended use or not:

"In the past four years [2017–2021] have also had a lot of patients express concern about the administration. And so wanting to kind of be as current as they can be with their devices and so potentially exchanging them sooner than they need."

- Complex Family Planning Specialist, West Coast, Academic Setting, always offers extended use.

Clinicians observed that patients are noticing and reacting to abortion restrictions when making their contraceptive decisions, which may impact the widespread implementation of extended use.

### Process of implementation

Many clinicians reported that a barrier to implementing extended use was patient preference for removal when they are already in clinic for a scheduled removal or replacement procedure, regardless of being counseled on extended use at that time:

“’Oh, I’m already here. I’m approved. Let’s just go ahead and get it done.’ So there’s probably not a whole lot you can do about that either, once they’re already in the clinic, and have their mind set on it.”

- Obstetrician-Gynecologist, Southeast, Academic Setting, does not offer extended use.

Many participants in this study noted that patients have made logistical arrangements prior to their appointments including paid time off, childcare, or prior authorization. It can be difficult for clinicians to offer extended use within this context, therefore counseling is better done prior to a patient coming in for a replacement appointment.

A perceived facilitator of extended use that was mentioned often was clear, concise clinician educational services or materials that illustrates existing data on efficacy and risks. Clinicians believed that this education could be in the form of continued medical education, targeted trainings, or written summaries of relevant studies, data, and recommendations. One consistency across interviews was that education on extended use must be integrated into regular practice and be easily understood by busy clinicians:

"I think that when we get a pamphlet or a brochure or a one page, something that just has everything condensed so it’s a really quick, oh, okay, this is something that we can be offering patients. And these are the reasons why it would be a benefit to them, and these are the patients that maybe would fall out of not offering this to. I think because of how busy we are, that’s the best way for us to make change."

- Advanced Practice Clinician, Southwest, Academic Setting, does not offer extended use.

Participants reported that these resources should be widely distributed beyond the complex family planning and obstetrician-gynecology community to increase accessibility to extended use.

Another potential facilitator identified was effective patient educational materials such as flyers that state the 5-year efficacy of the contraceptive implant, though producing these might require FDA approval. Participants in this study report that patients rely on clinicians to provide information on the efficacy and duration of their contraceptive implant. However, it is difficult for patients to accept extended use when there are inconsistencies across multiple sources of information:

"I mean, if online, there was information where it said you can keep it in for three to five years and they’re able to back that up. You know, people like to do their own research. I think that would be helpful, versus it says everywhere three, three, three, three, three, and then you’re the only person telling them something different, then it’s a little more tricky."

- Obstetrician-Gynecologist, West Coast, Community Setting, does not offer extended use.

Overall, participants in this study expressed that it would be helpful to have easily understood information for clinicians and patients that explained the evidence for extended use.

## Discussion

Our results demonstrate that there is an opportunity to increase widespread implementation of extended use through multiple interventions. Clinicians reported that patients prefer to have their implants replaced when they are already in clinic for the procedure. Therefore, intervening prior to replacement appointments at 3 years in the form of telemedicine visits or notifications from scheduling staff may make extended use of the contraceptive implant more acceptable to patients. Further, clinician and patient education on extended use that is easily understood and widely disseminated would likely increase use of the contraceptive implant up to 5 years.

The implementation of extended use of the contraceptive implant up to 5 years likely decreases healthcare costs secondary to fewer procedures and unintended pregnancies, and expands reproductive choices for patients seeking contraception. It has been found that clinicians who offer extended use state that most of their patients accept extended use when it is offered [14]. However, the reasons why a patient may or may not accept extended use are unclear, but may include changes in bleeding and concerns about use past the FDA-approved duration. Research on bleeding patterns in the extended use period may facilitate counseling and give patients a better expectation of possible changes they may see in years 4 and 5. Additionally, research on the patient perspective and acceptability of using the contraceptive implant past its FDA-approved timeframe is needed.

This study focused on clinicians and their perspectives on extended use. However, it is important to note that patients may be fully informed about extended use and choose to replace their implant at or before 3 years of duration. All discussions regarding contraception, including extended use of the implant, should always occur within a patient-centered and shared decision-making model. Widespread offering of extended use may allow for more patients to make fully informed decisions about the duration and use of their contraceptive devices, therefore expanding reproductive choice and agency in addition to potentially sparing patients from unnecessary procedures and extra healthcare costs.

Interestingly, although there are data to reflect high implant efficacy in years 4 and 5, [2–4] some participants in this study believe there is increased liability in counseling on off-label use without FDA approval. Importantly, off-label use is common among reproductive clinicians and is protected by the FDA if there is strong published evidence supporting off label use [5]. Additionally, the Society of Family Planning supports extended use of the contraceptive implant up to 5 years [22]. The FDA requires implant training for clinicians before they can insert or remove the implant. This training includes the FDA product labeling indicating the maximum duration of use for pregnancy prevention as three years [1]. It is possible that clinician training and product labels that advertise a 3-year duration dissuade clinicians from offering extended use of the contraceptive implant due to concerns about legal repercussions in the event of an unintended pregnancy with extended use. Therefore, organization- or systems-level guidelines, policy changes, and trainings in support of extended use may allow clinicians to feel comfortable offering off-label use of the implant. Additionally, FDA approval of the contraceptive implant to 5 years would likely greatly facilitate implementation of extended use.

Changing the FDA label to reflect extended use can be expensive, and contraceptive companies may not be incentivized to change the label. However, increasing the FDA approval of the contraceptive implant would allow for companies to have a longer-acting contraceptive device that is more directly comparable to other LARC devices such as the 52 mg LNG IUD that can be used for up to 8 years. If FDA approval for 5 years of use were to occur, it is not known if the barriers described in this study would continue to apply. However, it is likely that the facilitators of extended use from this study would support implementation of extended use irrespective of the federally approved duration.

One strength of the study is the national sample and the diversity of clinician types and settings. There is also representation of clinicians who consistently offer extended use and those who do not offer extended use. Another strength of this study is that it was designed utilizing a framework focusing on implementation, thus yielding results that can be used to create effective interventions.

Limitations of this study include the small sample size and selection bias from recruiting from a prior study that utilized listservs and social media. Additionally, we recruited from a population that was specifically interested in family planning and identified mostly as Caucasian and female. Because of this, our results may not be generalizable to the national population of clinicians who offer contraceptive implant services. However, our direct selection of participants who only sometimes or do not offer extended use allowed us to hear diverse perspectives regardless of prior knowledge or interest in extended use. Another limitation is that we did not ask advanced practice clinicians what their specific training was (i.e., nurse practitioner or physician’s assistant). As the training for advanced practice clinicians can vary greatly, our results may not be generalizable to all advanced practice clinicians.

## Conclusions

In conclusion, this study describes the barriers and facilitators to widespread implementation of extended use of the contraceptive implant. These results offer new perspectives and potential strategies to increase widespread implementation of extended use of the contraceptive implant up to 5 years of use. Based on our findings, there is opportunity to expand access to extended use by developing educational materials for clinicians and patients, identifying a champion of extended use, and counseling on extended use prior to removal appointments at 3 years. Of note, these results should be viewed in the context of recent policy access issues regarding reproductive health and used to support patient-centered contraceptive choices, regardless of a patient’s decision to extend use of their contraceptive implant up to 5 years. It is important that clinicians and patients utilize shared decision making when discussing extended use of the contraceptive implant.

### Supplementary Information

**Supplementary Material 1.**

## Data Availability

The datasets generated and/or analyzed during the current study are not publicly available due to being stored in a private, HIPAA-compliant database, but are available from the corresponding author on reasonable request.

## Abbreviations

CFIR: Consolidated Framework for Implementation Research
CFIR: Consolidated Framework for Implementation Research
FDA: Food and Drug Administration
IUD: CoIntrauterine device
LARC: Long-acting reversible contraception
LNG: Levonorgestrel
OBGYN: Obstetrician-Gynecologist
U.S.: United States

## References

[1] Nexplanon® Prescribing Information. Organon. 2021. https://www.organon.com/product/usa/pi_circulars/n/nexplanon/nexplanon_pi.pdf. Accessed 20 Feb 2023.

[2] McNicholas C, Swor E, Wan L, Peipert JF (2017). Prolonged use of the etonogestrel implant and levonorgestrel intrauterine device: 2 years beyond food and drug administration-approved duration. Am J Obstet Gynecol.

[3] McNicholas C, Maddipati R, Zhao Q, Swor E, Peipert JF (2015). Use of the etonogestrel implant and levonorgestrel intrauterine device beyond the U.S. food and drug administration-approved duration. Obstet Gynecol.

[4] Ali M, Akin A, Bahamondes L, Brache V, Habib N, Landoulsi S, Hubacher D, WHO study group on subdermal contraceptive implants for women (2016). Extended use up to 5 years of the etonogestrel-releasing subdermal contraceptive implant: comparison to levonorgestrel-releasing subdermal implant. Hum Reprod.

[5] U.S. Food and Drug Administration. (1998). *Off-label and investigational use of marketed drugs, biologics, and medical devices: guidance for institutional review boards and clinical investigators*. Retrieved from https://www.fda.gov/regulatory-information/search-fda-guidance-documents/label-and-investigational-use-marketed-drugs-biologics-and-medical-devices. Accessed 20 Dec 2022.

[6] Jensen JT, Lukkari-Lax E, Schulze A, Wahdan Y, Serrani M, Kroll R (2022). Contraceptive efficacy and safety of the 52-mg levonorgestrel intrauterine system for up to 8 years: findings from the mirena extension trial. Am J Obstet Gynecol.

[7] O’Dwyer MC. Contraceptive Efficacy of the Mirena Intrauterine System Through 8 Years of Use. NEJM Journal Watch. Retrieved from https://www.jwatch.org/na55371/2022/10/04/contraceptive-efficacy-mirena-intrauterine-system-through. Accessed 7 Mar 2024.

[8] Teunissen AM, Grimm B, Roumen FJ (2014). Continuation rates of the subdermal contraceptive Implanon(®) and associated influencing factors. Eur J Contracept Reprod Health Care.

[9] Moray KV, Chaurasia H, Sachin O, Joshi B (2021). A systematic review on clinical effectiveness, side-effect profile and meta-analysis on continuation rate of etonogestrel contraceptive implant. Reprod Health.

[10] Blumenthal PD, Gemzell-Danielsson K, Marintcheva-Petrova M (2008). Tolerability and clinical safety of implanon. Eur J Contracept Reprod Health Care.

[11] Mansour D, Korver T, Marintcheva-Petrova M, Fraser IS (2008). The effects of implanon on menstrual bleeding patterns. Eur J Contracept Reprod Health Care.

[12] Diedrich JT, Zhao Q, Madden T, Secura GM, Peipert JF (2015). Three-year continuation of reversible contraception. Am J Obstet Gynecol.

[13] Funk S, Miller MM, Mishell DR, Archer DF, Poindexter A, Schmidt J, Zampaglione E, Implanon US Study Group (2005). Safety and efficacy of implanon, a single-rod implantable contraceptive containing etonogestrel. Contraception.

[14] Rigler N, Averbach S, Sandoval S, Meurice M, Hildebrand M, Mody SK (2022). Barriers and facilitators of extended use of the contraceptive arm implants: a cross-sectional survey of clinicians. Obstet Gynecol.

[15] Hubacher D, Spector H, Monteith C, Chen PL (2018). Not seeking yet trying long-acting reversible contraception: a 24-month randomized trial on continuation, unintended pregnancy and satisfaction. Contraception.

[16] Winner B, Peipert JF, Zhao Q, Buckel C, Madden T, Allsworth JE, Secura GM (2012). Effectiveness of long-acting reversible contraception. N Engl J Med.

[17] Monea E, Thomas A (2011). Unintended pregnancy and taxpayer spending. Perspect Sex Reprod Health.

[18] CFIR Research Team-Center for Clinical Management Research. The Consolidated Framework for Implementation Research: Constructs. 2023. https://cfirguide.org/constructs/. Accessed 24 Jan 2023.

[19] Forman J, Damschroder LJ, Jacoby L, Siminoff L (2008). Qualitative content analysis. Empirical research for bioethics: A primer.

[20] Hill CE, Knox S, Thompson BJ, Williams EN, Hess SA (2005). Consensual qualitative research: an update. J Couns Psychol.

[21] Soratto J, Pires DEP, Friese S (2020). Thematic content analysis using ATLAS.ti software: potentialities for researchs in health. Rev Bras Enferm.

[22] Dethier D, Qasba N, Kaneshiro B (2022). Society of family planning clinical recommendation: extended use of long-acting reversible contraception. Contraception.

