# Supplementary material for: Offering extended use of the contraceptive implant via an implementation science framework: a qualitative study of clinicians’ perceived barriers and facilitators
Source: BMC Health Serv Res. 2024 Jun 3;24:697. doi: 10.1186/s12913-024-10991-4 (PMC11145768; doi:10.1186/s12913-024-10991-4)
Supplement: Supplementary file 1 — Supplementary Material 1. [file 12913_2024_10991_MOESM1_ESM.docx]

**Appendix A.** Interview Guide for Qualitative Study on Extended Use of the Contraceptive Implant

**Individual Characteristics**

*Patient Characteristics*

1. How would you characterize the patient population that your organization primarily serves, in terms of resource access and health literacy?

*Individual Stage of Change*

1. What is your experience and comfort in offering extended use of the implant?
2. *For providers who do not offer extended use:* What are the reasons that you do not offer extended use of the implant?

**Intervention Characteristics**

*Adaptability*

1. What kinds of changes do you think are necessary to make extended use of the implant work effectively in your setting, and who will decide whether these changes are needed?

*Complexity (only for those who offer extended use)*

1. How complicated is offering extended use of the implant?

**Outer Setting**

*Barriers:*

*Patient Barriers:*

1. What barriers have your patients faced in accessing and utilizing extended use of the implant?
2. From our survey with clinicians, the top patient barriers for accessing and utilizing extended use of the implant were: concerns about pregnancy risk, bleeding concerns, and patient preference for removal because they are already in clinic for replacement/ removal. Which of these has been the most challenging for your patients, and why?
3. Are there any additional patient barriers that we missed?

*Provider Barriers:*

1. What barriers have you as a clinician faced in offering extended use of the implant?
2. From our survey with clinicians, the top clinical challenges for offering extended use of the implant were: FDA approval, lack of educational resources for patients, concerns about liability, and lack of efficacy data for women with obese BMIs. Which of these has been the most challenging for you, and why?
3. Are there any additional clinical challenges that we missed?

*Available Resources*

1. Do you expect to have sufficient resources to implement offering extended use of the implant?
2. What resources do you need to address the patient barriers?
3. What resources do you need to address the clinical barriers?

*External Policies & Incentives*

1. What kind of financial or other incentives influence the decision to offer extended use?

*Facilitators:*

*Patient Facilitators:*

1. What factors have helped your patients access and utilize extended use of the implant?
2. From our survey with clinicians, the top patient factors for extended use of the implant were: prior counseling on extended use and patient request for extended use. In your experience, what is the impact of prior counseling on patients utilizing extended use?
3. Are there any other patient factors that you think would help patients utilize extended use?

*Provider Facilitators:*

1. What facilitators have you as a clinician faced in offering extended use of the implant?
2. From our survey with clinicians, the top clinical facilitators for offering extended use of the implant were: strong evidence supporting duration of use beyond FDA approval, colleagues offering extended use, training of schedulers and call center employees, and a champion of extended use at your institution. Which of these do you think would promote extended use of the implant the most?
3. Are there any additional clinical facilitators that we missed?

*Peer Pressure*

1. Who has the most important influence on you to offer extended use, and why?

**Inner Setting**

*Structural Characteristics*

1. What ideas do you have to facilitate implementation of extended use of the implant?
2. How do we make this happen? Who at your organization needs to be involved to implement these changes? (i.e., practice manager, clinic director, etc.)

*Champions*

1. Who are the champions of extended use, and what kinds of behaviors or actions do they exhibit?

*Costs/Concerns*

1. What concerns do you have about implementing extended use of the implant?

*Relative Priority*

1. What kinds of high-priority initiatives or activities are already happening in your setting?
2. What is the priority of implementing extended use of the implant relative to other initiatives that are happening now?

**Process of Implementation**

*Planning (only for those who do not offer extended use)*

1. What have you done (or what do you plan to do) to facilitate the implementation of offering extended use of the implant?
